# Effectiveness and safety of traditional Chinese medicine in the treatment of steroid-osteonecrosis of femoral head: A protocol for systematic review and meta-analysis

**DOI:** 10.1097/MD.0000000000026811

**Published:** 2021-07-30

**Authors:** Peilin He, Junming Chen, Chen Yue, Maoxiao Ma, Zhenqiang Hong, Youwen Liu

**Affiliations:** a Key Laboratory of Orthopedics & Traumatology of Traditional Chinese Medicine and Rehabilitation Ministry of Education (Fujian University of TCM), Fujian University of Traditional Chinese Medicine, Fuzhou; b Department of Orthopedic Surgery, Luoyang Orthopedic Hospital of Henan Province, Orthopedic Hospital of Henan Province, Luoyang; c Quanzhou Orthopedic-Traumatological Hospital Affiliated to Fujian University of Traditional Chinese Medicine, Quanzhou, China.

**Keywords:** protocol, steroid-induced osteonecrosis of the femoral head, systematic review, traditional Chinese medicine

## Abstract

**Background:**

Osteonecrosis of the femoral head (ONFH) is a common refractory disease in orthopedics. Overdose glucocorticoid application is a common trigger for ONFH. Traditional Chinese medicine (TCM), as a treatment for ONFH, has been shown to be effective in treating steroid-induced ONFH (SONFH). However, a systematic review and meta-analysis of them is lacking. We aim to systematically review the effectiveness and safety of TCM in the treatment of SONFH.

**Methods:**

We will search the following databases: PubMed, Embase, the Cochrane Library, MEDLINE, the Chinese Biomedical Literature Database, China Science and Technology Journal Database, China National Knowledge Infrastructure, and Wanfang Data (since the inception of the databases to the present). In addition, we will look for clinical trial registrations, prospective grey literature, relevant conference papers, and established study reference lists. We will use Review Manager 5.3 software for meta-analysis and heterogeneity assessment. We will evaluate the quality of the evidence using a hierarchy of recommendation assessment, development, and evaluation.

**Results:**

This study will systematically evaluate the efficacy and safety of TCM in the treatment of SONFH.

**Conclusion:**

This systematic review to evaluate the effectiveness and safety of TCM in the treatment of SONFH will provide updated evidence for clinical application.

**INPLASY registration number:**

INPLASY202170015.

## 1 Introduction

Osteonecrosis of the femoral head (ONFH) is a common and refractory disease in orthopedics.^[1,2]^ Excessive glucocorticoids (GCs) are a common cause of ONFH, and steroid-induced ONFH (SONFH) occurs in more than 40% of individuals who chronically overdose on GCs.^[3]^ SONFH occurs mainly in the young and middle-aged, aged 20 to 50 years, and without effective intervention, most patients develop femoral head collapse within 1 to 3 years after onset and have to undergo premature total hip arthroplasty.^[4]^ Clinical medical researchers believe that SONFH is a complication of hormone use, because long-term hormone use leads to poor body function, decreased circulatory system function, and slow blood flow, thus patients recover slowly and require a long treatment period, and the incidence of SONFH is significantly higher than that of traumatic osteonecrosis.^[5–10]^ To date, no specific drug has been developed clinically for the treatment of SONFH. In recent years, traditional Chinese medicine (TCM) has played an important role in the treatment of orthopedic diseases.^[11–13]^ There have been increasing numbers of clinical studies on SONFH prevention and treatment with TCM, and the efficacy is significant.^[14–16]^ It has been found clinically that TCM can achieve good results in SONFH treatment, and the results of a large number of animal experimental studies have shown that enhanced TCM use has good effects in SONFH treatment.^[17,18]^

There remains a lack of relevant systematic reviews in clinical practice, and the purpose of this protocol is to evaluate the efficacy and safety of TCM on SONFH based on the current studies.

## 2 Methods

### 2.1 Study registration

This protocol is registered with INPLASY (INPLASY202170015), which is based on the preferred reporting items for systematic review and meta-analysis protocol guidelines.^[19,20]^

### 2.2 Ethics and dissemination

Because the program does not require patient recruitment or the collection of personal information, no further ethical approval is required.

### 2.3 Inclusion criteria

#### 2.3.1 Types of studies

The included studies will be randomized controlled trials. There will be no restrictions on language or publication type. Nonrandomized clinical studies, quasi-randomized controlled trials, cellular experiments, animal experiments, cluster randomized controlled trials, and case reports will be excluded.

#### 2.3.2 Types of participants

The analysis will include participants who meet the diagnostic criteria for SONFH. All eligible study participants, regardless of age, race, or sex, will be included in this meta-analysis. Pregnant women, lactating women, and patients with severe diseases will be excluded.

#### 2.3.3 Types of interventions

Patients in the experimental group should have received TCM treatment alone or in combination with other treatment modalities. The species and combination methods of the TCM will not be considered. Patients in the control group will have received non-TCM treatments.

#### 2.3.4 Types of outcome measures

##### 2.3.4.1 Primary outcomes

Overall efficiency, hip pain score (visual analog scale score), and hip function (Harris score, WOMAC score, etc) will be the main outcomes.

#### 2.3.5 Secondary outcomes

1. Total hip replacement rate;
2. Progression rate of imaging staging of femoral head necrosis (staging methods: FICAT, ARCO, Japanese Joint Society staging);
3. Percentage of the volume of the femoral head necrosis.

### 2.4 Database search strategy

Electronic databases and other sources, including PubMed, Embase, the Cochrane Library, MEDLINE, the Chinese Biomedical Literature Database, China Science and Technology Journal Database, China National Knowledge Infrastructure, and Wanfang Data, will be searched using computer and manual methods. Different search methods will be adjusted according to different Chinese and English databases. We will briefly describe the search process for PubMed (Table 1).

**Table 1:** Search strategy for PubMed.

Number	Search terms
#1	Glucocorticoid-Induced Osteonecrosis of the Femoral Head[Title/Abstract]
#2	Glucocorticoid induced femoral head necrosis[Title/Abstract]
#3	femoral head necrosis[MeSH Terms]
#4	steroid-induced osteonecrosis of femoral head[MeSH Terms]
#5	femur head necrosis[MeSH Terms]
#6	steroid-induced femoral head necrosis[MeSH Terms]
#7	hormone femoral head necrosis[Title/Abstract]
#8	osteonecrosis[Title/Abstract]
#9	steroid necrosis of the femoral head[Title/Abstract]
#10	hormonal avascular necrosis of femoral head[Title/Abstract]
#11	steroid osteonecrosis of the femoral head[Title/Abstract]
#12	or/#1-#11
#13	chinese herbal medicine[All Fields]
#14	traditional chinese medicine[MeSH Terms]
#15	chinese medicine[All Fields]
#16	herbs[All Fields]
#17	chinese herbs[All Fields]
#18	herbal[All Fields]
#19	traditional medicine[All Fields]
#20	or/#13-#19
#21	randomized controlled trial [Title/Abstract]
#22	controlled clinical trial [Title/Abstract]
#23	randomized [Title/Abstract]
#24	randomised [Title/Abstract]
#25	placebo [Title/Abstract]
#26	randomly [Title/Abstract]
#27	trial [Title/Abstract]
#28	groups [Title/Abstract]
#29	or/#21-#28
#30	#12and#20and#29

### 2.5 Data management selection process and data items

**Figure 1:**
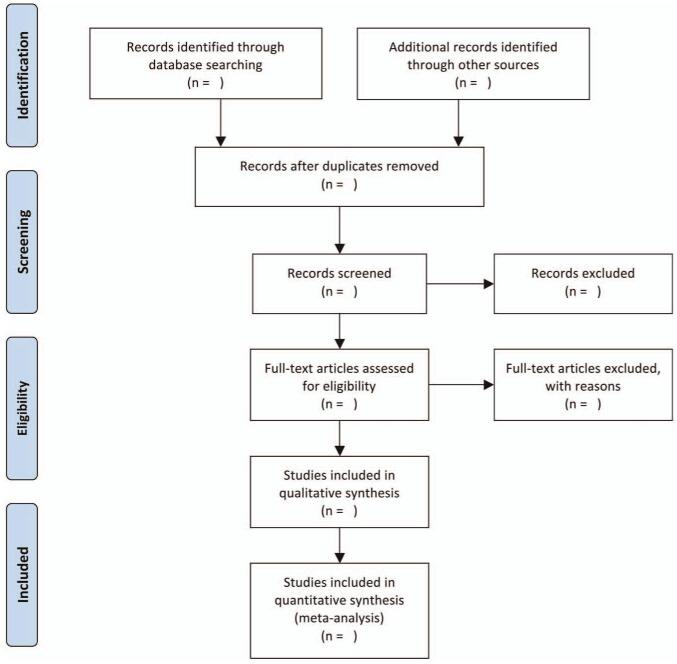
PRISMA flow diagram. PRISMA = preferred reporting items for systematic reviews and meta-analysis.

Two researchers will independently search all relevant literature, first reading the titles and abstracts of the literature to eliminate duplicate content. Eligible studies will then be imported into EndNoteX8 software for management. Finally, the full text will be read to identify eligible studies. If any disputes arise, the 2 researchers will discuss and reach an agreement. When a consensus cannot be reached, disagreements will be resolved with the assistance of a third investigator. If any literature is found to be missing, it will be added by contacting the original author. The study selection process will strictly follow the PRISMA flowchart. (Fig. 1).

Two researchers will independently complete the research assessment based on the eligibility assessment form and extract general information about the selected articles. Included information will be first author, sex, publication year, country, study design, sample size, detailed interventions, safety, outcomes, disease duration, and follow-up time. The above information will be repeatedly reconciled by 2 investigators, and conflicts regarding data extraction will be resolved by consensus.^[21,22]^

### 2.6 Risk of bias will be assessed in the included studies

Two investigators will assess the risk of bias based on the Cochrane Handbook for systematic reviews 5.3 (https://www.cochrane.org/). Recommended assessment tools will independently assess methodological quality. In case of disagreement, this will be resolved by discussion between the 2 reviewers or with the assistance of a third investigator.

### 2.7 Statistical analysis methods

We will use RevMan 5.3, provided by the Cochrane Collaboration, for data analysis. We will use the Chi-square test and *I*^*2*^ statistic to determine heterogeneity between studies. We consider heterogeneity between studies to be low when *P* ≥ .05 and *I*^*2*^ ≤ 50%. We will use fixed-effects models for the statistics. When *P* < .05 and *I*^*2*^ > 50%, we will consider the heterogeneity between studies to be high. We will use a random-effects model for the statistics. All data analyses will be conducted with 95% confidence intervals. Continuous data will be analyzed as the mean difference or the normalized mean difference, whereas dichotomous data will be examined as the relative risk. When *P* < .05, it indicates that the difference is statistically significant. If the heterogeneity between studies is high, we will perform subgroup analysis on different herbal medicines to explore whether herbal medicines cause heterogeneity. In addition, a sensitivity analysis will be performed if necessary.^[23]^

### 2.8 Assessment of reporting biases

If the number of included studies exceeds 10, we will use funnel plots to measure publication bias. The results of the funnel plot will also be interpreted in detail.

## 3 Discussion

SONFH is one of the most difficult diseases to cure in orthopedics. Many patients with ONFH eventually require hip replacement because of irreversible collapse of the femoral head, increased pain, and hip dysfunction. The prevalence of SONFH can reach 9% to 40% in patients receiving long-term and excessive hormone therapy, and its main prevalent population consists of the young and middle-aged. In most cases, the disease affects both femoral heads at the same time, leading to a high rate of disability and causing great difficulties for families and society.^[24,25]^ GCs are widely used in medical procedures because they are inexpensive and effective, but they also have side effects that must be considered. Many patients suffer irreversible damage to the femoral head because of overdose or prolonged use of glucocorticosteroids, which can have a significant impact on patients.^[26]^

In recent years, an increasing number of studies have demonstrated the effectiveness and safety of TCM in the treatment of SONFH, but high-quality systematic and scientific evaluations are lacking. The purpose of this study will be to evaluate the effectiveness and safety of TCM in the treatment of SONFH. In addition, we hope to promote the development and application of TCM for the benefit of patients.

## Author contributions

**Conceptualization:** Peilin He, Youwen Liu.

**Data curation:** Peilin He, Junming Chen, Chen Yue, Maoxiao Ma, Zhenqiang Hong, Youwen Liu.

**Formal analysis:** Peilin He, Junming Chen, Chen Yue.

**Methodology:** Peilin He, Junming Chen, Chen Yue.

**Project administration:** Youwen Liu.

**Supervision:** Youwen Liu.

**Writing – original draft:** Peilin He, Junming Chen.

**Writing – review & editing:** Peilin He, Junming Chen, Chen Yue, Maoxiao Ma, Zhenqiang Hong, Youwen Liu.

**Conceptualization:** Peilin He, Youwen Liu.

**Data curation:** Peilin He, Junming Chen, Chen Yue, Maoxiao Ma, Zhenqiang Hong, Youwen Liu.

**Formal analysis:** Peilin He, Junming Chen, Chen Yue.

**Methodology:** Peilin He, Junming Chen, Chen Yue.

**Project administration:** Youwen Liu.

**Supervision:** Youwen Liu.

**Writing – original draft:** Peilin He, Junming Chen.

**Writing – review & editing:** Peilin He, Junming Chen, Chen Yue, Maoxiao Ma, Zhenqiang Hong, Youwen Liu.
